# miRModuleNet: Detecting miRNA-mRNA Regulatory Modules

**DOI:** 10.3389/fgene.2022.767455

**Published:** 2022-04-12

**Authors:** Malik Yousef, Gokhan Goy, Burcu Bakir-Gungor

**Affiliations:** ^1^ Department of Information Systems, Zefat Academic College, Zefat, Israel; ^2^ Department of Computer Engineering, Faculty of Engineering, Abdullah Gul University, Kayseri, Turkey; ^3^ The Scientific and Technological Research Council of Turkey, Ankara, Turkey

**Keywords:** gene expression, multi omics, machine learning, integrative “omics”, feature selection

## Abstract

Increasing evidence that microRNAs (miRNAs) play a key role in carcinogenesis has revealed the need for elucidating the mechanisms of miRNA regulation and the roles of miRNAs in gene-regulatory networks. A better understanding of the interactions between miRNAs and their mRNA targets will provide a better understanding of the complex biological processes that occur during carcinogenesis. Increased efforts to reveal these interactions have led to the development of a variety of tools to detect and understand these interactions. We have recently described a machine learning approach miRcorrNet, based on grouping and scoring (ranking) groups of genes, where each group is associated with a miRNA and the group members are genes with expression patterns that are correlated with this specific miRNA. The miRcorrNet tool requires two types of -omics data, miRNA and mRNA expression profiles, as an input file. In this study we describe miRModuleNet, which groups mRNA (genes) that are correlated with each miRNA to form a *star shape*, which we identify as a miRNA-mRNA regulatory module. A scoring procedure is then applied to each module to further assess their contribution in terms of classification. An important output of miRModuleNet is that it provides a hierarchical list of significant miRNA-mRNA regulatory modules. miRModuleNet was further validated on external datasets for their disease associations, and functional enrichment analysis was also performed. The application of miRModuleNet aids the identification of functional relationships between significant biomarkers and reveals essential pathways involved in cancer pathogenesis. The miRModuleNet tool and all other supplementary files are available at https://github.com/malikyousef/miRModuleNet/

## 1 Introduction

The World Health Organization (WHO) reported in 2019 that cancer is the leading cause of death in three out of four countries in the world (Sung et al., 2021). Approximately 19.3 million people were diagnosed with cancer in 2020 and 10 million people lost their lives due to cancer. Lifestyles, environmental, demographic and cultural factors all contribute to these problematic statistics. If these statistics are to change, it is important to better understand the complex molecular processes that lead to cancer development and progression as precisely as possible. This information is critical to both traditional drug development approaches and for personalized medicine approaches (Schmidt, 2014).

miRNAs are non-coding RNAs, roughly 22–25 nucleotides in length (Bartel, 2004; Allmer and Yousef, 2016; Allmer and Yousef, 2022) and are present in animals and plants, as well as in humans. The observations that miRNAs with similar sequences are detected in all living things further support the idea that miRNAs perform critical biological functions (Cai et al., 2009). miRNAs are known to be responsible for the regulation of approximately 60% of protein coding genes (Friedman et al., 2009) and cellular processes including cell proliferation, apoptosis and necrosis (Keller et al., 2011). miRNAs can affect gene expression by binding to the seed regions of mRNAs (Ivey and Srivastava, 2015; Yousef et al., 2018) and, in general, repress the expression of their target mRNAs via physically interacting with them. In other words, miRNAs tend to have a negative correlation with mRNAs. The elucidation of the relationships between miRNAs and mRNAs is important in order to understand the mechanisms of complex diseases such as cancer (Pencheva and Tavazoie, 2013; Yousef et al., 2014). A better understanding of the associations between miRNAs and the mRNAs can reveal important information on normal and aberrant gene regulation and cell biology.

**FIGURE 1:**
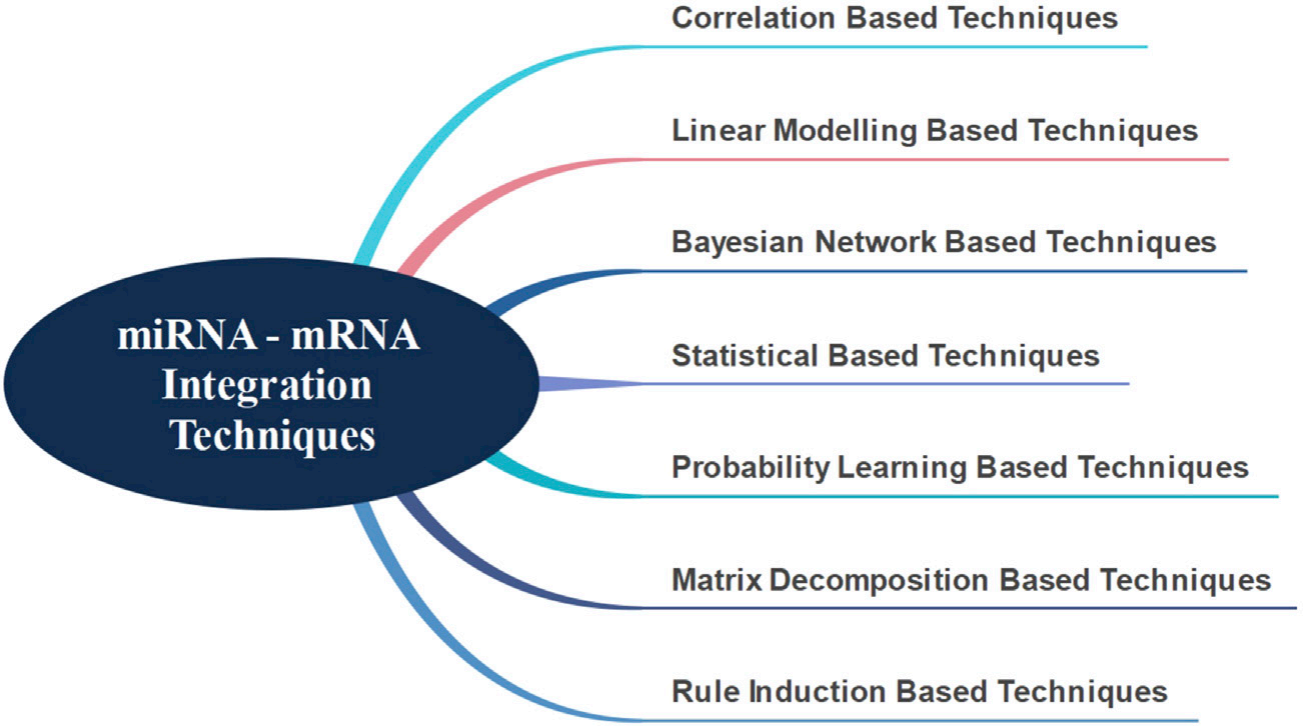
miRNA - mRNA integration techniques.

There are presently seven major techniques in literature for the integration of miRNA-mRNAs, as shown in Figure 1 (Masud Karim et al., 2016). In general, the correlation-based techniques primarily start by identifying differentially expressed mRNAs and miRNAs. Using various correlation metrics, mRNA-miRNA pairs are identified and the integration is achieved through these pairs (Feng et al., 2018; Li et al., 2018; Liu et al., 2018; Yang et al., 2019; Yao et al., 2019). Hailu et al. (2021) have used Spearman’s correlation and attempted to identify target genes and signaling pathways associated with pediatric dilated cardiomyopathy by integrating miRNA and mRNA data.

Correlation-based techniques have the following disadvantages. These techniques assume that one miRNA affects only one mRNA, an assumption that is not entirely true (Huang et al., 2007). Linear modeling based techniques have been developed in order to overcome this assumption. Huang et al. (2007) suggested modeling mRNA expressions as linear combinations of miRNAs to address this problem and applied the Bayesian algorithm to discover hidden miRNA targets. They also used a different distribution technique, integrating sequence information with their previous study. Stingo et al. (2010) proposed a comparable approach. However, they did not consider the effect of different tissues and suggested that miRNAs had a different promoter effect on each mRNA (Le and Bar-Joseph, 2013) attempted to find the mRNA modules that affect the functionality of miRNAs, using interaction, expression and sequence information; and a regression-based solution. They claimed that by using this method, they could identify relevant modules in a more robust and accurate way.

Another approach used for the integration of miRNA and mRNA interactions is the Bayesian network technique. Liu et al. (2009) performed an integrated analysis using differentially expressed miRNAs and mRNAs through Bayesian network technique. Due to the large amount of biological data available, (Madadjim, 2021) emphasized the necessity of producing a scalable solution and suggested that the Bayesian network-based machine learning model could be a valid solution.

All events that take place in a living system happen within a specific biological organization. In other words, the events that occur at the molecular level are not random. This understanding has motivated the development of statistical solutions for miRNA and mRNA integration (Jayaswal et al., 2011). Along this line, (Hecker et al., 2013) evaluated different miRNA-mRNA expression data using statistical approaches, without any other prior knowledge; and developed a method to distinguish different tissues. Using a similar approach, (Nersisyan et al., 2021) developed a new tool to generate miRNA-gene-TF networks.

Another method that generates miRNA-mRNA groups is the probability learning based technique. In this approach, the interaction probabilities of known miRNA-mRNA pairs are estimated (Joung et al., 2007). However, in order for this operation to be performed robustly and effectively, more than one source of information is needed. The Non-Negative Matrix Factorization technique is another important method. This method accomplishes the integration process by successfully separating different information sources (Zhang et al., 2011) was able to successfully integrate information obtained from different sources and generate significant miRNA-mRNA groups. Additional approaches use rule induction-based techniques based on information theory. Generally, as in the other techniques, data obtained from more than one data source needs to be integrated (Tran et al., 2008) used a rule induction-based technique to find miRNA-mRNA groups while (Lavrac et al., 2004) used the CN2-SD system as the rule generation system to identify miRNA-mRNA groups.

With the advancements in technology we now have access to data which describes different levels of molecular regulation from the same individual. These rich and complicated data sets require the development of novel techniques to integrate and understand this data. All the tools that we have surveyed above are based on statistical approaches. To the best of our knowledge, there are only two available tools that can adequately address the classification problem using integrated miRNA-mRNA groups. These bioinformatics tools are maTE (Yousef et al., 2019) and miRcorrNet (Yousef et al., 2021b). The main difference between these two tools is the miRNA-mRNA grouping methodology. While maTE adopts a biological grouping methodology, miRcorrNet tool uses correlation information in order to generate the groups. These two tools not only solve the classification problem, but also provide a score for each group, where the score reflects the contribution of each group to classification.

In this study, we present a novel bioinformatics tool named miRModuleNet. miRModuleNet differs from our two previous approaches in that miRNA-mRNA integration has been developed using statistical information. In this paper, we have comparatively evaluated these three different grouping methodologies and showed the superiority of miRModuleNet against state of the art methods.

## 2 Materials and Methods

### 2.1 Datasets

In this study, miRNA and mRNA expression profiles which have been obtained from the same individuals have been used. Due to the aforementioned reasons, in this study we focused on cancer. In this context, 11 different cancer datasets were downloaded from The Cancer Genome Atlas (TCGA) data portal (Tomczak et al., 2015). The details of these datasets are presented in Table 1.

**TABLE 1:** Details of the datasets utilized in miRModuleNet.

TCGA data	Abbreviation	Control	Case	PMID
Bladder urothelial carcinoma	BLCA	405	19	24476821
Breast invasive carcinoma	BRCA	760	87	31878981
Kidney chromophobe	KICH	66	25	25155756
Kidney renal papillary cell carcinoma	KIRP	290	32	28780132
Kidney renal clear cell carcinoma	KIRC	255	71	23792563
Lung adenocarcinoma	LUAD	449	20	25079552
Lung squamous cell carcinoma	LUSC	342	38	22960745
Prostate adenocarcinoma	PRAD	493	52	26544944
Stomach adenocarcinoma	STAD	370	35	25079317
Papillary thyroid carcinoma	THCA	504	59	25,417,114
Uterine corpus endometrial carcinoma	UCEC	174	23	23636398

Control and case columns denote the number of samples. Column PMID refers to Pubmed ID of the related publication, where further information about the dataset can be found.

### 2.2 The G-S-M Approach

miRModuleNet was developed based on the generic approach named G-S-M. This generic approach was adopted by different tools such as SVM RCE, SVM-RCE-R (Yousef et al., 2007; Yousef et al., 2021a), maTE (Yousef et al., 2019), CogNet (Yousef et al., 2021d), miRcorrNet (Yousef et al., 2021b), and Integrating Gene Ontology Based Grouping and Ranking (Yousef et al., 2021c). Recently, these tools and their competitors were reviewed in (Yousef et al., 2020).

**FIGURE 2:**
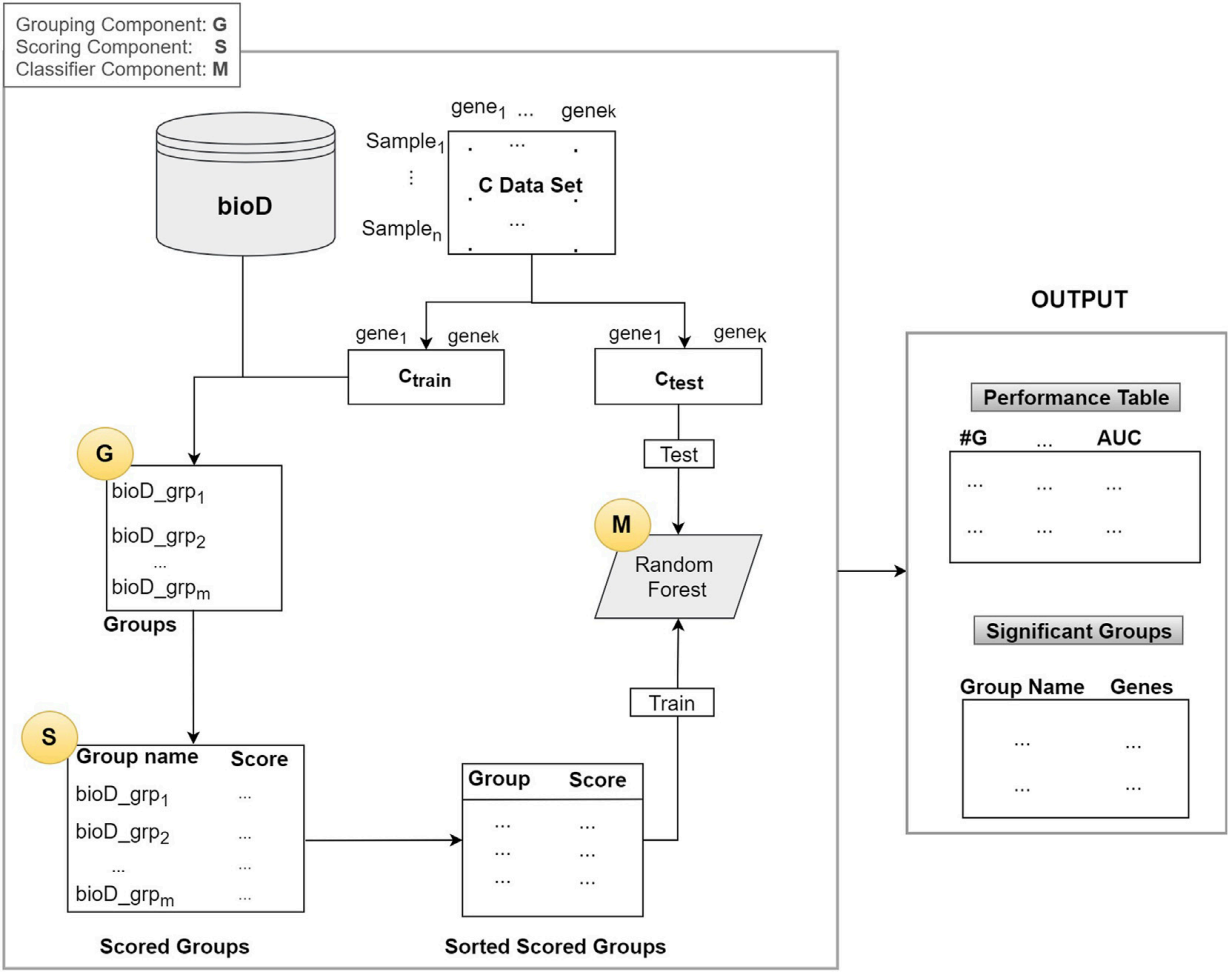
The general integrative approach that is based on grouping and scoring/ranking.

As illustrated in Figure 2, the algorithm mainly consists of 3 components (shown as circles):

1. G Component: Detect groups of genes
2. S Component: Score the groups.
3. M Component: Creates the model by training a classifier (Random Forest)

In the first component G, bioD is a biological database, or another prior biological knowledge that will be used to create the *groups* that contain the genes from the mRNA (gene expression) data. This operation is represented as the G component whose output is the set of groups. Group names are the names of the biological entity such as miRNA names, where a group of genes may be targeted by that miRNA, a KEGG pathway name, or a disease phenotype name. Note that, in most of the cases each group has an important biological meaning. The resulting set of groups is indicated in the Groups box in Figure 2.

Assume that we have n samples and k genes in our dataset C. The C data is split into two parts as C_train_ and C_test_, where the C_train_ is used to score the groups and to train the classifier in the M component. The C_test_ is used for testing and reporting the performance.

Let m = *size*(Gr) be the number of groups generated by the G component and let Gr be the collection of all the groups as Gr = [bioD_grp_f_, where *f = 1,..,size*(Gr)]. From now on, we will refer to one group of Gr as *bioD_grp*.

**FIGURE 3:**
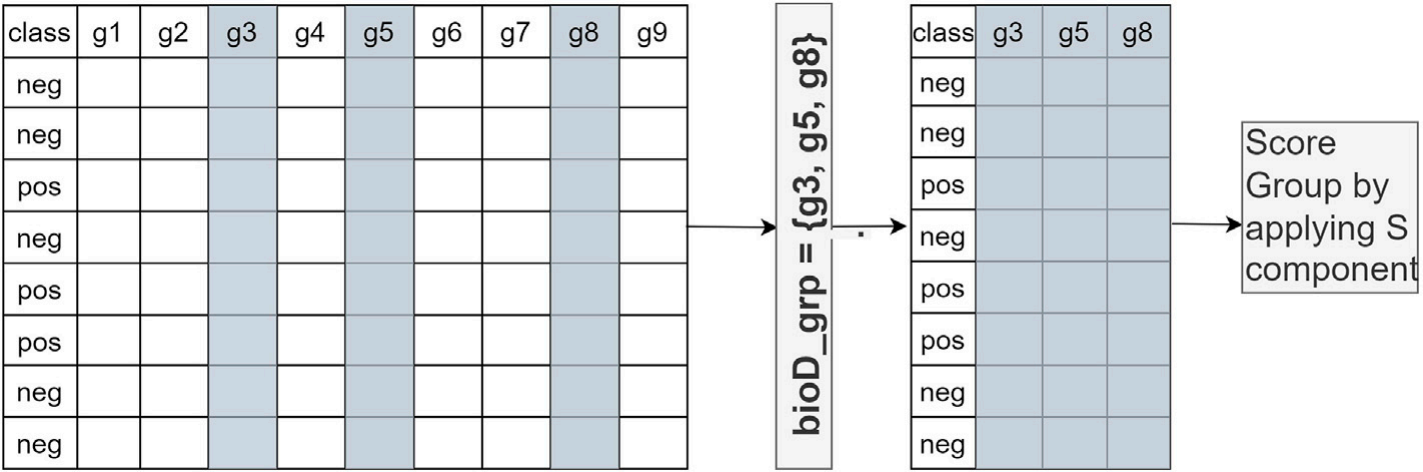
An example of how to create sub_data based on a group of genes and then this sub-data is subject to the scoring component S. g_i_ refers to gene_i_.

In Component S, each *bioD_grp* in Gr is scored, as shown in Figure 2. In order to perform this task, we generate *size* (Gr) different sub_data sets which are the sub matrices of the gene expression matrix C_train_ (illustrated in Figure 2). Each sub_data set includes the columns from the original data matrix C_train_, corresponding to the genes in *bioD_grp*. In other words, each sub_data set contains only the gene expression values of specific genes included in that group and associated class labels. We will refer to each sub_data as C_train_sub_f_, where *f* = 1,..,size(Gr) that contains genes that belong to the group of *bioD_grp*. Figure 3 is an example of how to create sub_data based on a group of mRNAs and then this sub-data is subject to a procedure for scoring those groups.

Let S (sub_data) be the k-fold cross validation procedure that computes and returns some performance measurements such as accuracy, specificity, sensitivity and Area Under the ROC Curve (AUC). We used AUC as the major performance metric to score for the sub_data. Next, we score all the groups using the S function which produces scores for groups, named as grp_scores and grp_scores = [(bioD_grp_f_, score_f_) *f = 1,..,size*(G)]*.* Then we sort this list based on score and obtain grp_scores_sorted = [(bioD_grp_sorted_f_, score_sorted_f_) f = 1,..,size(G)]. Table 2 presents an example output of this S component. In Table 2, microRNAs are shown as the group name since in this example miRNAs are used within the G component to group a set of genes targeted by that miRNA.

**TABLE 2:** A sample output of scoring component when applied on THCA data, downloaded from TCGA.

Group	Accuracy	Sensitivity	Specificity	FM	Precision	Cohen’s kappa
hsa-miR-101-3p	0.89	0.82	0.92	0.85	0.88	0.73
hsa-miR-200c-3p	0.95	0.92	0.97	0.92	0.94	0.89
hsa-miR-508-3p	0.98	0.93	1.00	0.96	1.00	0.94
hsa-miR-629-5p	0.99	0.97	1.00	0.98	0.99	0.97

Each miRNA ID represents a group, which is generated by the Grouping Component G. Groups are sorted according to the accuracy metric.

The last component is the M component, which creates the model by training a classifier. In order to build the Random Forest (RF) model and report the cumulative performance of the algorithm, we implement the procedure presented in Table 3. Here, *top*_*f*_ specifies the number of top groups defined by the user*.*

**TABLE 3:** Pseudocode of component M, which calculates the performance.

For *f* = 1 to *top*_*f*_
genes_set = $\;\overset{f}{\underset{j=1}{\cup }}$ {bioD_grp_sorted_j_}
X_train = sub_set of C_train_ that includes the genes from the genes_set
X_test = sub_set of C_test_ that includes the genes from the genes_set
RF_Model < - train Random Forest (X_train)
Performances = test RF_Model (X_test)

In Table 3, RF_Model is the model created by training Random Forest on the X_train data set. This model will be used to test on the X_test. In Table 3, grp_bioD_sorted_f_ is one of the groups of Gr (for example, of miRNA, KEGG, GO databases). The Performance Table in Figure 2 describes the cumulative performance of the G-S-M approach, where *#G* is the number of genes in the cumulative group. The output of this step is the Performance Table shown in the right hand side of Figure 2.

### 2.3 miRModuleNet

**FIGURE 4:**
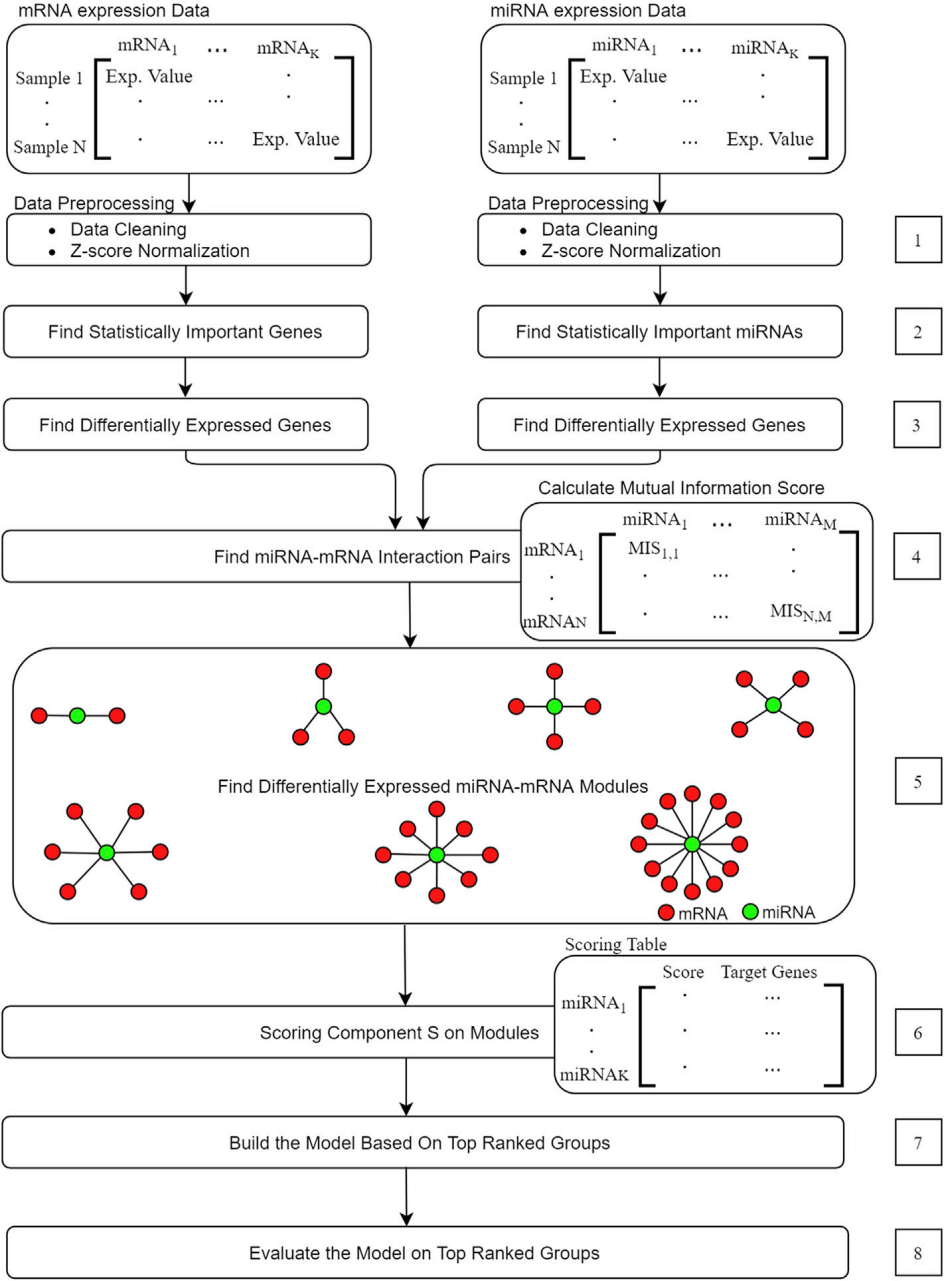
miRModuleNet flowchart.

miRModuleNet tool is developed as a specific application of our G-S-M approach on the -omics data integration problem including miRNA and mRNA expression profiles. Hence, miRModuleNet makes use of the above-mentioned G-S-M approach with further additions. Before utilizing the G-S-M method, miRModuleNet includes some preprocessing steps as explained in detail below. The main idea behind miRModuleNet is illustrated in Figure 4. Initially, both miRNA and mRNA expression datasets are split into training and testing parts. Following the general idea presented in Figure 2, the training part is used to create the groups, define the features in each group and to build the model, while the testing part is only considered in the evaluation step.

In the 1^st^ step of miRModuleNet, both miRNA and mRNA expression profiles are cleaned by removing the columns containing the missing data. For miRNA-seq profiles, raw read counts were normalized to reads per million mapped reads (RPM). For mRNA-seq profiles, the raw read counts were normalized to Reads Per Kilobase Million Mapped Reads (RPKM). Subsequently, whole data at different ranges were normalized using z-score normalization. Second step identifies statistically important miRNAs and mRNAs that were to be used in the following steps. In the 3^rd^ step, using statistically significant miRNAs and mRNAs, differentially expressed miRNAs and mRNAs are detected using the edgeR package (Robinson et al., 2010). In step 4, the mutual information matrix is generated in order to determine the miRNAs and mRNAs that will be used to form the miRNA-mRNA groups. Instead of considering each pair in this matrix, we only select the pairs that exceeded a certain threshold. We experiment with the values of 0.15, 0.25, and 0.5 as the Mutual Information (MI) threshold and present data identifying the value of 0.25 as the optimal threshold value. This value is used in the later steps of miRModuleNet. The 5^th^ step corresponds to the grouping component in the general approach. In this step the miRNA-mRNA regulatory groups i.e., modules are generated according to the Algorithm 1. Here, I(x,y) denotes the mutual information between two variables x and y. I(x,y) = H(x)−H(y|x), where H(y) and H(y|x) are the entropy of y and the conditional entropy of y given x. The strategy for obtaining miRNA-mRNA regulatory modules is explained in the following section.

Algorithm 1 Generate the “Star shaped” module that contains single miRNA and multiple mRNAs.

1) Let C= {gene1, gene2, ...genek } be the profiles of the mRNAs from data Dgenes
2) Let Str ←∅ be the “Star” group for the miRNA
3) Compute I_i_ = I(gene_i_, miRNA) of each mRNA gene_i_ in C.
4) Let gene*= max_i_ {I_i_}, Select the gene with the highest value of mutual information

### 2.4 Generating the miRNA-mRNA Regulatory Modules/Groups

In order to detect the miRNA-mRNA regulatory modules, we have used the RFCM^3^ approach suggested by (Paul and Madhumita, 2020). The RFCM^3^ considers two types of -omics data, the miRNA and mRNA expression profiles from the same samples. Here, we will use the terms module and groups interchangeably. miRNA-mRNA modules consist of a miRNA and its related mRNA genes. As illustrated in the Step 5 of Figure 4, we have generated the module called the star shaped module, where it contains a single miRNA and multiple mRNAs/genes. As suggested by (Paul and Madhumita, 2020), mRNAs for such modules are selected in such a way that they are simultaneously and functionally similar to the corresponding miRNA.

In creating these groups, we first identify the miRNA-mRNA pair with the highest score. As shown in green in Step 5 of Figure 4, we detect the center of the star (the miRNA that serves as the group name). The mRNA in this pair is the starting point for the addition of other mRNAs forming the star shape. The relationship of the miRNA to other mRNAs is determined by looking at the Mutual Information matrix. For mRNAs to be included in the group, the mutual information score between them and the relevant mRNA must exceed the threshold set by the end user and this relationship is then considered to be potentially important.

The 6^th^ step corresponds to the scoring component S in the general approach. In this step, the classification power of each group is evaluated by calculating the scores, which indicate how powerful a group is in terms of distinguishing the two classes (case/control). At the end of this step a Scoring Table is produced containing the miRNA in rows and the score of the corresponding mRNA group in the columns. In the 7^th^ step, a machine model is trained using the top ranked groups. In other words, the machine learning model which uses Random Forest is trained via only considering top *f* groups. This means that miRModuleNet is using all of the genes within top *f* groups in a unified manner. The default value of *f* is set as 10 and miRModuleNet generates 10 different machine learning models where each model is trained using a different number of groups from 1 to 10. The user can also change the value of the *f*. Classification strategy is explained more in detail in the following section. Then the last step is the evaluation step that uses the test part.

### 2.5 Classification Approach

In this study, the Random Forest algorithm (Breiman, 2001), which is a supervised machine learning algorithm, was used to solve the classification problem. This algorithm consists of two stages. In the first stage, a forest is created by producing a large number of decision trees. In the second stage, the classification process is carried out through the feedback obtained from these trees. As an advantage of this use, a model with better generalization can be produced. On the one hand, a more robust solution is obtained, on the other hand, overfitting is potentially prevented.

While generating the model, 100 fold Monte Carlo Cross Validation (MCCV) was used in the learning phase (Xu and Liang, 2001). In order to evaluate the performance, miRModuleNet repeats the process 100 times. In each iteration, 90% of the data is selected for training and the remaining 10% is selected for testing. In addition, an under sampling method was used to solve the imbalanced class problem encountered while training the model. This method aims to provide the desired rate of data distribution by randomly eliminating samples from the class with too many samples. Hence, miRModuleNet randomly selects samples with a ratio of 1:2 for under-sampling. Under-sampling was performed in every iteration of cross validation. In each iteration, our approach generates lists of miRNA modules/groups and their associated genes that are slightly different. Hence, there is a need to apply a prioritization approach on those lists. As utilized in miRcorrNet, we have used rank aggregation methods. In this respect, we have embedded the RobustRankAggreg R package, developed by (Kolde et al., 2012) into miRModuleNet workflow. The RobustRankAggreg assigns a *p*-Value to each element in the aggregated list, which describes how good each element/entity was ranked compared to the expected value.

### 2.6 Implementation of miRModuleNet

**FIGURE 5:**
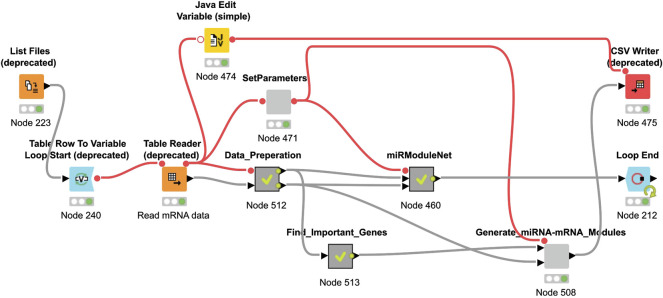
miRModuleNet workflow in KNIME.

The KNIME Analytics platform is used for the implementation of miRModuleNet (Berthold et al., 2008). The KNIME environment is easy to use, it is an open source platform and it can be used for a wide variety of operations and for a wide variety of data types. In the KNIME environment, all operations work based on workflows. miRModuleNet’s workflow is shown in Figure 5.

As it can be seen in Figure 5, KNIME workflows consist of nodes, where each of these nodes perform a specific task. For example, using the List Files node, the directory where the data is located is specified. By using the Table Reader node, it is ensured that the data is imported into the KNIME environment. By using the Data Preparation metanode, above-mentioned preprocessing operations are performed. miRModuleNet metanode is the node of the main algorithm. In addition to these, within the SetParameters node, two critical parameters of the workflow can be entered by the end user. These parameters are the number of iterations and the mutual information threshold.

Results are obtained after running the KNIME workflow, which is shown in Figure 5. One of these results is the comparison of the performances of the machine learning models depending on the k (number of top groups) parameter. An example of this comparison is shown in Table 4. Table 4 presents an example performance table of miRModuleNet for top ranked 10 modules for BLCA data. The last row presents the performance of the top ranked module/group (#Groups = 1). In other words, an accuracy of 89% is obtained using 79.25 genes on average. The row of #Groups = 2 presents the performance metrics obtained for the top 2 groups where the genes of the top ranked group and the second highest scoring group are aggregated together. That is to say that miRModuleNet reports the performance results for top 10 groups cumulatively.

**TABLE 4:** An example performance table of miRModuleNet for top ranked 10 modules for BLCA dataset.

#Groups	#Genes	Accuracy	Sensitivity	Specificity	AUC
10	1422.96	0.92	0.89	0.94	0.98
9	1254.76	0.92	0.88	0.93	0.98
8	1110.82	0.91	0.87	0.93	0.97
7	962.83	0.91	0.88	0.93	0.97
6	799.7	0.92	0.88	0.94	0.97
5	628.14	0.92	0.87	0.94	0.97
4	489.59	0.91	0.87	0.93	0.98
3	331.02	0.90	0.85	0.93	0.97
2	205.08	0.90	0.84	0.93	0.97
1	79.25	0.89	0.82	0.92	0.95

## 3 Results

### 3.1 Performance Evaluation Metrics

The performance of machine learning models can be evaluated through several quantitative metrics. In this respect, statistical metrics such as Accuracy, Sensitivity, Specificity and Precision could be calculated by constructing the confusion matrix. For the problems involving imbalanced data, it is essential to prove the consistency of the results. In this regard, Area Under the Curve (AUC) metric is reported as an accurate metric in terms of evaluating the performance results in such problems (Hand, 2004).

### 3.2 Performance Results

#### 3.2.1 Optimization of Mutual Information Threshold

**FIGURE 6:**
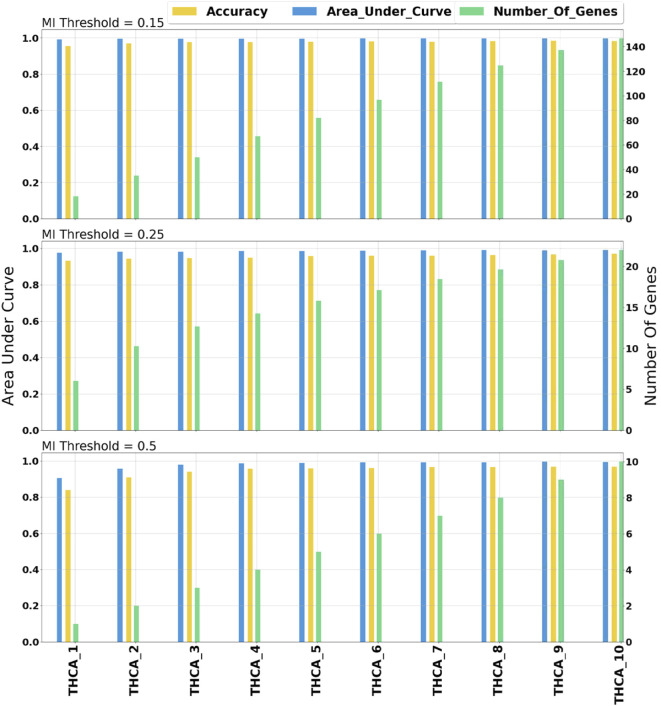
Comprehensive evaluation of different mutual information threshold values. The numbers following the underscore values correspond to the number of groups.

miRModuleNet tool uses (MI) to detect the relationships between miRNAs and mRNAs. In order to identify the optimal value of the MI threshold, we experimented with three different values (0.15, 0.25, 0.5). As stated above, we selected 0.25 as the optimal threshold. In our comparison, the AUC value versus the number of genes is taken into account. Such a comparison on THCA data is demonstrated in Figure 6. As illustrated in Figure 6, when the MI threshold value was set to 0.15, the AUC value was in the range of 0.98–0.99, and the number of genes increased from 18 to 146 as the number of groups (star shaped modules) increased. Using the MI threshold value as 0.25, AUC values in the range of 0.97–0.99 were obtained, and the number of genes increased from 6 to 22. When the MI threshold value was set to 0.5, the AUC value was in the range of 0.92–0.99, and the number of genes increased from 1 to 10. Such comparisons were made for all cancer types. As a result of these comparative evaluations, we have decided to set the MI threshold as 0.25.

In this study, we have tested miRModuleNet using 11 different cancer datasets presented in Table 1. Our machine learning models generate the most important group as an output; and the performance evaluation metrics were obtained by using the identified most important group. As presented in Table 5, the average number of selected genes for the most important groups was 38.27 for 11 tested cancer types. Likewise, the average of obtained AUC values using the top group was 0.98. All performance results reported in this study were obtained by calculating the mean of the 100-fold Monte Carlo Cross Validation (MCCV).

**TABLE 5:** Performance results of miRModuleNet over the top-ranked group.

Disease	#Genes	ACC	SEN	SPE	FM	AUC	Precision
BLCA	79	0.89	0.82	0.92	0.85	0.95	0.88
BRCA	22	0.95	0.92	0.97	0.92	0.98	0.94
KICH	40	0.98	0.93	1.00	0.96	0.99	1.00
KIRC	64	0.99	0.97	1.00	0.98	0.99	0.99
KIRP	41	1.00	0.99	1.00	0.99	1.00	1.00
LUAD	4	0.94	0.90	0.96	0.90	0.98	0.93
LUSC	12	0.98	0.99	0.98	0.98	1.00	0.97
PRAD	5	0.86	0.76	0.91	0.77	0.92	0.82
STAD	115	0.90	0.81	0.95	0.85	0.97	0.92
THCA	6	0.93	0.90	0.95	0.90	0.98	0.92
UCEC	33	0.94	0.89	0.96	0.89	0.99	0.94

ACC stands for Accuracy, SEN stands for Sensitivity, SPE stands for Specificity, FM stands for F-Measure, AUC stands for Area Under the ROC curve.

In addition, in terms of performance, miRModuleNet has been compared with other existing tools i.e., SVM-RFE, maTE and miRcorrNet. These tools differ in terms of the data they use and the way they produce results. While miRcorrNet and miRModuleNet both use miRNA and mRNA expression profiles, SVM-RFE and maTE tools use only mRNA data. In addition, while miRcorrNet, miRcorrNet and maTE give the results on group level, the SVM-RFE tool gives the results directly at the gene level. In other words, miRcorrNet, maTE and miRModuleNet tools give their results by building a Random Forest model over the top 1 to 10 cumulative groups of genes. On the other hand, SVM-RFE tool gives its results using different levels of genes, i.e., 1, 2, 4, 6, 8, 10, 20, 40, 60, 80, 100, 125, 250, 500 and 1,000 genes. In order to make a fair comparison of the existing methods involving different approaches, it has become necessary to determine benchmarks at both the group level and the gene level. The comparison level for miRcorrNet, miRModuleNet and maTE, which produced results at the group level, was determined as two according to the number of genes criterion. When these three tools used two as the group level, the lowest number of genes was found to be 7.48, and the highest used number of genes was found to be 141.26. Therefore, it was decided to use gene levels 8 and 125 to be able to include the SVM-RFE tool in the comparison. In Table 6, the performance evaluation of all these tools are presented. The calculated performance metrics are number of genes, accuracy, sensitivity, specificity, F-Measure, AUC and Precision. Table 6 indicates that miRModuleNet achieved a similar performance by using nearly half of the genes compared to another newly developed tool called miRcorrNet. Although there are no serious differences in results, the increase in the AUC metric is considered to be very important and noteworthy. Additionally, the close performances of the tools show that the developed tool miRModuleNet is a consistent and robust tool.

**TABLE 6:** Comparative evaluation of existing tools using 11 cancer datasets.

Method	#Genes	Accuracy	Sensitivity	Specificity	AUC	SD
miRModuleNet	78.31	0.96	0.91	0.98	0.99	0.04 ± 0.02
miRcorrNet	141.26	0.96	0.94	0.97	0.98	0.05 ± 0.05
maTE	7.48	0.96	0.94	0.96	0.98	0.034 ± 0.02
SVM-RFE	8	0.84	0.85	0.85	0.91	0.07 ± 0.04
SVM-RFE	125	0.96	0.97	0.95	0.98	0.05 ± 0.03

AUC column refers to the area under the curve values. All the presented values are average values over 100 MCCV for the level of top 2 groups for miRModuleNet, maTE and miRcorrNet; 8 and 125 genes for SVM-RFE. Standard Deviation (SD) values are given for AUC.

### 3.3 Functional Enrichment Analysis Results

**FIGURE 7:**
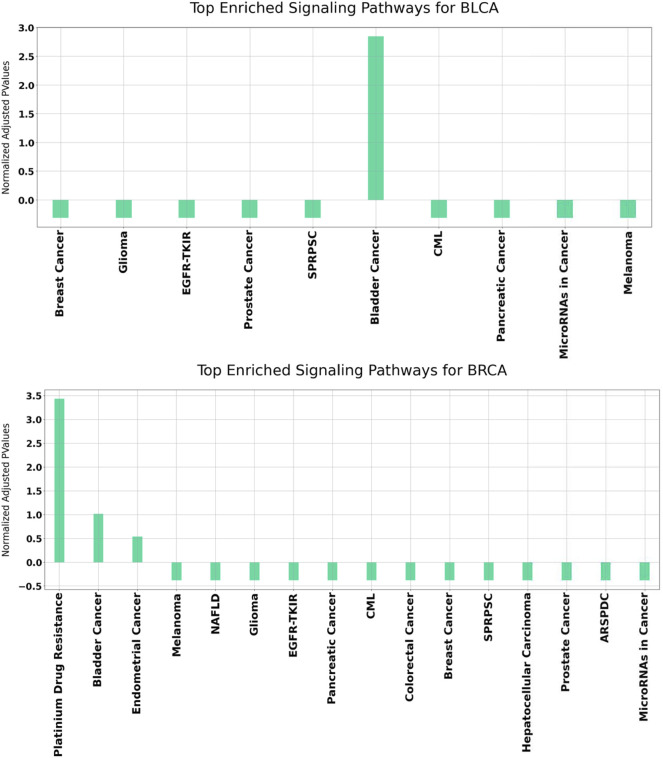
Functional enrichment results for BLCA and BRCA using GeneCodis. The *p* Values of the enriched KEGG pathways refer to the normalized values using mean normalization. SPRPSC stands for Signaling Pathways Regulating Pluripotency of Stem Cells, EGFR-TKIR stands for EGFR Tyrosine Kinease Inhibitor Resistance, CML stands for Chronic Myleoid Leukemia, NAFLD stands for Non-Alcoholic fatty Liver Disease, ARSPDC stands for AGE-RAGE Signaling Pathway in Diabetic Complications.

In order to better understand the disrupted mechanisms of the disease at the molecular level, functional enrichment analysis was carried out. Hence, we investigated whether the obtained results have biological meaning. For this purpose, GeneCodis (Tabas-Madrid et al., 2012) and DAVID (Huang et al., 2009a; Huang et al., 2009b), which have been widely used in literature, are utilized. For each disease, all enriched KEGG pathways were found separately. Overrepresented KEGG pathways of our identified set of genes in BLCA and BRCA datasets are presented in Figure 7.

**FIGURE 8:**
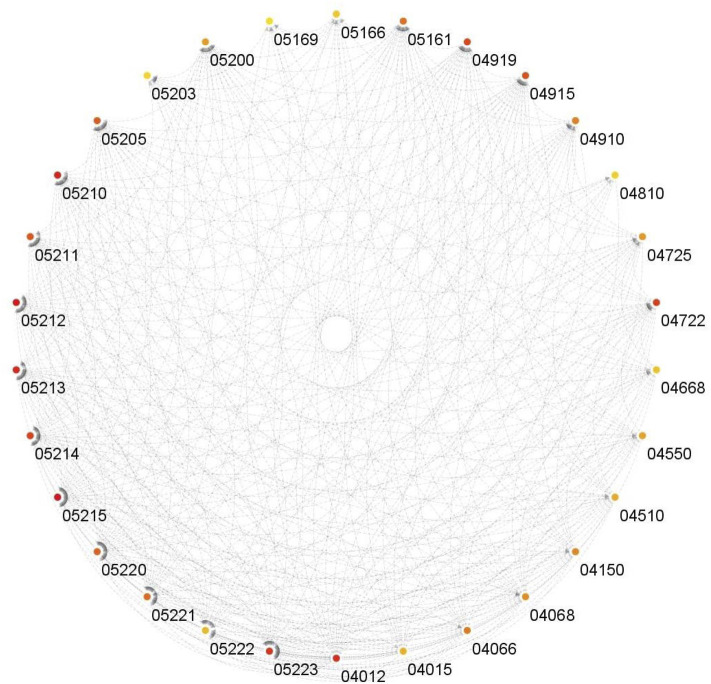
Interactions of the commonly overrepresented pathways in all datasets.

It can be observed from Figure 7 that for both BLCA and BRCA, the overrepresented pathways are directly related to the specific cancer types. We also felt that it was important to determine the pathways affecting different cancers and, we carried out additional procedures to better understand the molecular level relational networks of cancer. Using DAVID, we found that 55 pathways were commonly enriched in all the cancers tested. For these 55 pathways, a pathway - pathway interaction network was generated using the method that was developed in (Goy et al., 2019). A pathway network was obtained by examining the commonalities among the genes of the overrepresented pathways. Kappa statistics were used as distance metric. In order to construct a pathway - pathway interaction network, 3,025 pairwise relationships were analyzed for 55 commonly overrepresented pathways for 11 cancer types. To be able to find biologically relevant pairs, we used a Kappa score threshold. In this way, we aimed to keep only the interaction pairs, which are considered to be statistically important in terms of understanding the mechanisms of diseases at the molecular level. When this threshold was set as 0.15, the number of pathway pairs decreased to 403. The cytoHubba plugin (Chin et al., 2014) in the Cytoscape (Shannon et al., 2003) was used to detect the most important nodes in this pathway-pathway interaction network and Matthews Correlation Coefficient (MCC) values of each node (pathway) were calculated. We observed that 30 of the 55 pathways had very high MCC scores (between E^14^ and E^30^). The constructed pathway-pathway interaction network is presented in Figure 8.

### 3.4 Validation of miRModuleNet’s Results Using External Data

In order to check the robustness and reliability of miRModuleNet, an external dataset was considered. In this context, the GSE40419 dataset (Seo et al., 2012) was downloaded from the Gene Expression Omnibus database (Barrett & Edgar, 2006). The GSE40419 dataset was derived from 87 lung carcinoma cases and 77 normal people not having the disease. In this study, we refer to this dataset as LUSC_E. In our validation experiments, while the TCGA LUSC data is used as a train set, the LUSC_E dataset is used as a test set. To this end, we have used another KNIME workflow, which is developed for this type of tests. This workflow has also been added as a supplementary material.

Testing was carried out as follows. All genes for specific diseases in the train data and significant genes obtained by miRModuleNet are kept in separate files. To make a fair comparison, the number of random and significant genes was determined as 1, 5, 30, and 50. Subsequently, using the test KNIME workflow, the results were obtained both using these random genes and using the significant genes found by miRModuleNet. While the accuracy obtained using only 1 random gene was 51%, the accuracy reached 87% when the most important 1 gene found by miRModuleNet was used. Likewise, when comparing 50 genes, accuracy increased by approximately 11% with miRModuleNet, and reached 95%. Summary of these results are shown in Table 7. It can be concluded from Table 7 that miRModuleNet is robust, reliable and noteworthy. Moreover, the performance for the training data (TCGA LUSC) is also presented as a supplementary file.

**TABLE 7:** Performance results on the external validation data.

Experiments using different gene levels (1–5–30–50)	Sensitivity	Specificity	Accuracy	F-measure
Random 1 gene	0.43	0.58	0.51	0.44
Top 1 gene of mirModuleNet	0.84	0.88	0.87	0.85
Random 5 genes	0.46	0.61	0.55	0.48
Top 5 genes of mirModuleNet	0.94	0.81	0.88	0.87
Random 30 genes	0.57	0.91	0.76	0.68
Top 30 genes of mirModuleNet	0.94	0.92	0.93	0.92
Random 50 genes	0.76	0.94	0.86	0.83
Top 50 genes of mirModuleNet	0.94	0.97	0.95	0.94

In all experiments, the model is trained on TCGA- LUSC data and tested on external data, which is LUSC_E.

## 4 Discussions

### 4.1 Biological Interpretation of the Results

In bioinformatics problems, the biological value that the tool is providing is as important as the comparative performance evaluation with existing tools. In this section, we explore those features and provide a biological validation of our tool.

### 4.2 Validation of miRModuleNet’s Results on miRNA-Disease Association Databases

miRModuleNet produces multiple files as an output. One of these output files is the list of significant miRNA groups that are predicted to have a relationship with the disease and the genes targeted by these miRNAs. In the output file, these miRNAs are sorted according to their *p*-Values, which are assigned by the RobustRankAggreg method. In order to show the biological relevance of our findings, we refer to the miRNA - Disease association databases that are widely used in the literature. These databases are HMDD (Huang et al., 2019), miR2Disease (Jiang et al., 2009), miRcancer (Xie et al., 2013), dbDEMC (Yang et al., 2010) and PhenomiR (Ruepp et al., 2010). For each disease, miRNAs which were scored high in miRModuleNet and have *p*-Value less than 0.05 were checked in these databases to see whether there was a known relationship with the disease under study. Table 8 presents the comparison of the miRNAs identified for Lung squamous cell carcinoma (LUSC) against these five databases. This table displays the identified miRNA, its *p*-Value and the databases in which the miRNA is known to be associated with the relevant disease. For 11 different cancer datasets, a total of 682 miRNAs were found to be important by miRModuleNet. Among these selected miRNAs, approximately 34% of them were found in only one database, 23% were present in 2 databases, 15% in 3 databases, 10% in 4 databases, and 6% in 5 databases and 75 of the identified miRNAs were not listed in any of the databases. The details are presented in Table 9.

**TABLE 8:** Biological validation of the identified miRNAs for LUSC data by miRModuleNet, against five disease databases, i.e., dbDEMC, miRcancer, miR2Disease, PhenomiR, HMDD.

miRNA	Score (*p*-value)	Source(s)
hsa-miR-181a-5p	4.83E-58	dbDEMC, miRcancer, PhenomiR
hsa-miR-126-5p	2.79E-57	dbDEMC, miRcancer, miR2Disease, PhenomiR, HMDD
hsa-miR-140-3p	5.9E-55	dbDEMC, miRcancer, miR2Disease, PhenomiR, HMDD
hsa-miR-708-5p	5.9E-55	dbDEMC, miRcancer
hsa-miR-195-5p	5.9E-55	dbDEMC, miRcancer, miR2Disease, PhenomiR, HMDD
hsa-miR-30d-5p	7.76E-53	dbDEMC, miRcancer, PhenomiR,HMDD
hsa-miR-30a-5p	7,76E-53	dbDEMC, miRcancer, miR2Disease, PhenomiR, HMDD

**TABLE 9:** Summary of the comparison against the databases of miRNA–disease associations.

Disease	Number of miRNA-disease associations identified by miRModuleNet	Number of databases containing the specific miRNA—disease association				
		1	2	3	4	5
BLCA	62	21	17	9	6	2
BRCA	51	4	15	19	11	—
KICH	61	34	15	—	—	—
KIRC	46	27	9	5	—	—
KIRP	87	44	19	4	—	—
LUAD	91	11	26	31	15	8
LUSC	54	2	6	10	15	20
PRAD	53	9	11	14	13	4
STAD	35	8	14	6	4	2
THCA	55	28	9	8	2	4
UCEC	87	46	20	—	—	—

The numbers in the table indicate the number of identified miRNA–disease associations included in 1, 2, 3, 4, or 5 different databases.

It is very difficult to develop a sound machine learning model for diseases such as cancer, which have complex molecular mechanisms. In order to overcome this challenge, it is crucial to integrate different types of -omics data. Hence, effective machine learning models that provide reliable results need to be developed. To this end, in this study we aimed to develop a robust machine learning model that can classify the samples as cancer patients and controls via integrating miRNA and mRNA expression profiles. A variety of studies have been reported that use either mRNA or miRNA data alone or in combined fashion. Some studies are only presented as methods and others as publicly available tools. However, most of the existing tools are limited in use and, to the best of our knowledge, are web based and R based. MMIA (Nam et al., 2009), MAGIA (Sales et al., 2010), miRConnX (Huang et al., 2011) originally offered as web servers and are currently not available. anamiR (Wang et al., 2019) and miRComb (Vila-Casadesús et al., 2016) which are offered as R packages, cannot be used with the latest versions of R.

In comparison, the miRModuleNet has a user-friendly structure and is evaluated on 11 different cancer datasets. In addition, although we focused on a biological problem in miRModuleNet, the same approach can be adapted to any classification problem including two dimensional data. This is not the case with most of the models listed above. miRModuleNet KNIME workflow generates different output files. These outputs provide information about identified mRNAs, miRNAs and their groupings. The mRNAs, miRNAs and mRNA-miRNA groups that were considered to be potentially important were identified and all results were validated using the following two methods. The first is a literature based validation of the miRNA - disease relationships that were predicted by the miRModuleNet using five widely used databases, i.e., dbDEMC, miRcancer, miR2Disease, PhenomiR, HMDD. The second method is validation using an independent external dataset that was not included in training. Such experiments evaluate whether the generated model can be utilized on a totally independent cohort. Our findings using four different levels (1, 5, 30 and 50 genes) imply that miRModuleNet maintains good performance metrics when applied to new independent data.

## 5 Conclusion

Exploring the biological functions of differentially expressed genes through the integration of different types of -omics data such as miRNA and mRNA expression profiles remains an important research topic. However, the problems associated with how to best assess the repression effect on target genes using integrated miRNA/mRNA expression profiles are not fully resolved. To address this problem, we have proposed a novel tool, miRModuleNet, which conducts a machine learning-based integration of two-omics datasets to detect miRNA-mRNA modules that are most significant to the classification task. The tool detects the miRNA/mRNA groups, which are later subjected to Rank procedure. The strength of miRModuleNet is that the identified set of genes that are represented in groups are guaranteed to distinguish two classes (cases vs. controls) and may serve as a biomarker for the specific disease under investigation.

## Data Availability

The datasets presented in this study can be found in online repositories. The names of the repository/repositories and accession number(s) can be found in the article/Supplementary Material.
